# Analysis of IPV success treatment from an AI approach

**DOI:** 10.1371/journal.pone.0323945

**Published:** 2025-06-03

**Authors:** Isabel María Benjumeda Wynhoven, Claudio Córdova Lepe

**Affiliations:** 1 Facultad de Artes Liberales, Universidad Adolfo Ibáñez. Campus Viña del Mar, Valparaíso, Chile; 2 Interdisciplinary Center for Biomedical Research and Health Engineering. University of Valparaiso, Valparaíso, Chile; University of Ghana College of Humanities, GHANA

## Abstract

Intimate partner violence (IPV) is a serious social problem in Chile. Understanding the patterns of internalization and the motivations maintaining it is crucial to design optimal treatments that ensure adherence and completeness. This, in addition, is essential to prevent revictimization and improve the quality of life of both victims and their children.The present study analyzes the success of a psychological treatment offered by a Chilean foundation helping IPV victims. A database analysis containing 1,279 cases was performed applying classical statistics and artificial intelligence methods. The aim of the research was to search for cluster grouping and to create a classification model that is able to predict IPV treatment completeness. The main results demonstrate the presence of two main clusters, one including victims who completed the treatment (cluster 1) and a second one containing victims who did not complete the treatment (cluster 2). Cluster classification using an XGBoost model of the treatment completeness had an accuracy of 81%. The results showed that living with the aggressor, age and educational level had the greatest impact on the classification. Considering these factors as input variables allow for a higher precision on the treatment completeness prediction. To our knowledge, this is the first study performed in Chile that uses AI for cluster grouping and for analyzing the variables contributing to the success of an IPV victims’ treatment.

## Introduction

Intimate partner violence (IPV) constitutes a serious public health problem and a violation of human rights [1,2]. Globally, around one in three women between 15 and 49 years of age have suffered physical and/or sexual violence at some point in their lives [3]. Any woman is vulnerable to suffering IPV at some point in her life [4–6].

Multiple studies explored the damage that IPV produces both physically and psychologically [7,8]. The psychological costs of IPV are multiple and complex and include depression, anxiety, panic attacks, feelings of hopelessness, low self-esteem, chronic pain, and even suicide [9,10]. Women who suffer IPV are socially and cognitively affected [11,12], and their children often develop violent behavior, impaired self-esteem and academic performance [13].

Latin America presents very high rates of IPV [14,15]; According to previous studies, IPV prevalence ranged from 1% in Canada to 27% in Bolivia, confirming that nearly one-third (29.8%) of ever-partnered women in Latin America and the Caribbean have ever been physically and/or sexually abused by an intimate partner [16].

In Chile, 4 out of 10 women had suffered psychological violence by their partner: 1 in 4, physical violence; and 1 in 10, sexual violence, according to the National Women’s Service [17] [Labra-Valerdi., 2021].

However, IPV is still considered a private issue that must be solved at home [18,19]. Cultural beliefs, in addition to fear, economic and emotional dependence contribute to maintain IPV [20–22].

The Chilean context is in a serious mental health crisis [23]. Chilean women reported worse levels of self-perceived health during the pandemics [24] and Chile is in an alarming situation in terms of IPV [25]. In Chile, the intrafamily violence indicator has increased significantly over the last years, from 32.6 percent in 2012 to 41.4 percent in 2020 [26] (Saavedra et al., 2022]. Data analyzed after the first wave of Covid-19 lockdown in Latin America showed that women were more affected than men in mental health, with Chile leading the worst mental health indicators [27] (Salas Quijada et al., 2023]. Compared to other Latin American countries such as Brasil or Argentina, mental health in Chile has worsened dramatically in recent years, with women leading in anxiety, depression, and stress [28] [Duarte & Jimenez-Molina, 2022].

This situation is expected to increase due to the elevated rates of female unemployment [29,30]. Together with this, mental health problems, lacking social support and living with the aggressor are risk factors to suffer and maintain IPV [17].

The absence of psychological support in IPV victims increases the risk of post-traumatic stress disorder and impairs the quality of life of women and their children [31,32]. Therefore, receiving therapy and support is crucial for recovery and for a normal and healthy life [33,34]. Studies related to IPV and mental health interventions on IPV survivors are limited, as well as research analyzing the success rate of those interventions [35]. As a consequence, treatment programs are limited and not designed following a local perspective [17].

Chilean women often postpone their self-care for taking care of their family [14]. According to the ecological perspective, IPV is a complex phenomenon combining individual, family, community, and national characteristics [36][Flake and Forste, 2006]. IPV is more common in those countries presenting a patriarchal code that positions women in a submissive condition [37,38][Arce-Rodríguez, 2006, Romero y González, 2017]. Gender based norms reinforce male authority and superiority over females in most Latin American countries. In this context, machismo is often used to describe Latino masculinity, and refers to the cultural expectation that males must be masculine, strong, and sexually aggressive [39][Malonda et al., 2022]. In this scenario, the male dominance is accentuated by the expected role of women as submissive, dependent and sexually faithful to their husbands [36,40][Flake and Forste, 2006; López-Alvarado et al., 2020].

This may explain treatment abandonment and the lack of legal orientations demands, which in turn contributes to IPV relapse. Therefore, analyzing the variables leading to treatment abandonment is crucial to design adequate treatments that heal and restore IPV victims.

To our knowledge, this is the first study that deeply analyzes the variables affecting the success of an IPV treatment. The results presented here will contribute to the design of more successful and contextualized programs addressing IPV.

## Methodology

### Research design and participants

A quantitative, non-experimental, descriptive-analytical study was conducted with a transversal cut. The data used for this study was obtained from the Cicatrices Foundation. The Cicatrices foundation exists since march 2021 and is devoted at helping IPV victims, offering psychological support, as well as legal and social orientation in Valparaíso, Chile. The foundation offers free help and operates online and so it reaches the whole country. The analyzed sample was 1,279 Chilean women suffering IPV who were users of the foundation from 2021 to 2023. The exact dates when data were accessed for research purposes were 3^rd^ of October 2023, and different days in November and December 2023. The data was stored in a platform owned by the same foundation and was exported through an xlsx file containing no personal information such as name or address. The file contained sensitive information related to variables such as age, number of children, region, living with the aggressor, having support network, taking medications and education level. Whether users needed social or legal referral was also recorded, as well as closing observations (whether they completed treatment or not). Users gave consent for their personal information to be used for research purposes when they filled in the online form asking for help at the foundation.

### Instrument description

In order to receive help, victims filled in an online form and were contacted by a trained social worker via telephone. In this first contact, a structured questionnaire was used to interview the victim. Questions asked were name, place of residence, phone number, age, number of children, working outside, type of work, medication intake, cohabitation with the aggressor, having support network and what type and education level. All this information was stored in a database owned by the Foundation (Sirus) in which the application date was stored, as well as the date of creation of the record (starting date of the intervention).

Program description: After the first interview, a member of the psychological team of the Foundation contacted the victim and both agreed on the date and type of contact (zoom, meet, video call or normal call). Confidential management of the personal information was assured, and all the process was backed up in a database with a summary of each psychological session, and the dates. The Foundation offers a total of four free psychological sessions during which the victim talks about her case and the psychologist in charge accompanies her in the process of identifying the problem, the available networks, the victim´s strengths, and her options to leave the IPV situation. After finishing the four sessions treatment, the IPV victim was given the opportunity for continue with the same psychologist with the possibility of obtaining a discount on the sessions. The foundation offered legal and social orientation as well, if needed. Legal orientation refers to legal procedures related to violence complaints, separation or divorce from the aggressor or alimony and was provided by a lawyer. On the other hand, social orientation refers to guidance related to house rentals, aids or subsidies from the state. This help was provided by a trained social worker.

### Variables analized

Data was collected using a structured questionnaire applied by a trained psychologist or social worker from the Institution during a first interview. The form contained questions labeled as dichotomic variables (yes/no) such as whether the IPV survivor took any medication (in general, duration was not specified), if she was living with the aggressor, if she was working outside the house, if she had network support and if she finished the 4 psychological sessions included in the treatment. Other variables were the survivor´s age and the number of children (quantitative). Finally, educational level was measured as a categorical value (0 no education, 1 basic education, 2 middle education, 3 technical education and 4 university). When the survivor finished the treatment (if she did), the date was specified as well as if she required social and/or legal orientation/referral. If the victim did not finish the treatment, the reasons for abandoning were registered.

### Descriptive statistics

Statistical analysis and graph presentation correspond to a parametric analysis. The normality of the data was checked using the Shapiro Wilk test and the means were analyzed using ANOVA and pairwise comparison using T-test with a significance level of 95%. Frequency graphs are presented as bars of the grouped absolute values and mean graphs as boxes and SDs. All analysis and graphs were performed with Python 3.0 with the spicy stats, matplotlib and seaborn libraries.

### Group analysis using hierarchical clustering

The cleaned data was imported into a dataframe operable through Python 3, using the Jupyter Notebook code IDE. Subsequently, hierarchical clustering was applied using the Scipy libraries, applying a cut in the first division of the dendrogram and assigning the labels corresponding to the assigned group in a new ‘Cluster’ column. Subsequently, the comparison of variables by cluster was carried out applying ANOVA and Chi2_contigency tests. The box and bar graphs used to represent the variables by cluster were built using the Seaborn libraries.

#### Treatment completeness classification model using explainable machine learning.

A binary classification model of the therapy completeness was trained based on scikit learn’s train_test_split library. To split the data into training and test with a ratio of 0.75:0.25. For this, 50 randomization state seeds were tested to find the model with the best performance measured with precision. Subsequently, the SHAP libraries were used for the explainability of the trained machine learning model, where the impact of each variable on the output was represented with a swarm graph.

## Results

### Clustering

**Fig 1:**
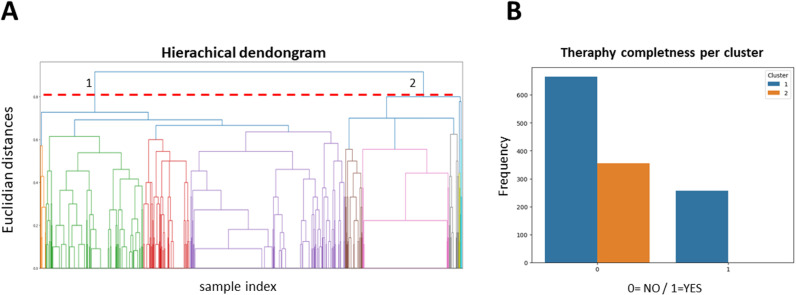
Clustering and Treatment completeness. (A). Relative euclidean distances calculated for the hierarchical clustering. The first data division in two big groups of different heterogeneity and amount is observed at a distance of 0.8. (B). Treatment completeness frequency. Treatment completeness frequency (1) versus not completeness (0) according to the cluster. Chi square was used to establish cluster and treatment completeness relations, showing a significant association with a p value of 2.4^−28^.

The analyzed database contained 1,279 cases of IPV victims that were divided by the program into two clusters through hierarchical clustering (Fig. 1): Cluster 1: N = 831 cases and Cluster 2: N = 329 cases, both with an obvious separation and with a Euclidean distance of 0.83. (Fig. 1A). The first difference with a significant association with the cluster was treatment completeness, where the users who did not complete the treatment were labeled as 0 and included those who never started and those who abandoned in intermediate stages. On the other hand, users who completed the treatment were labeled as 1. The results showed that 1/3 of the patients in cluster 1 completed the treatment, while in cluster 2 no one completed it. Association between the cluster and treatment completeness was statistically significant (Fig. 1B).

### Variables association per cluster

**Fig 2:**
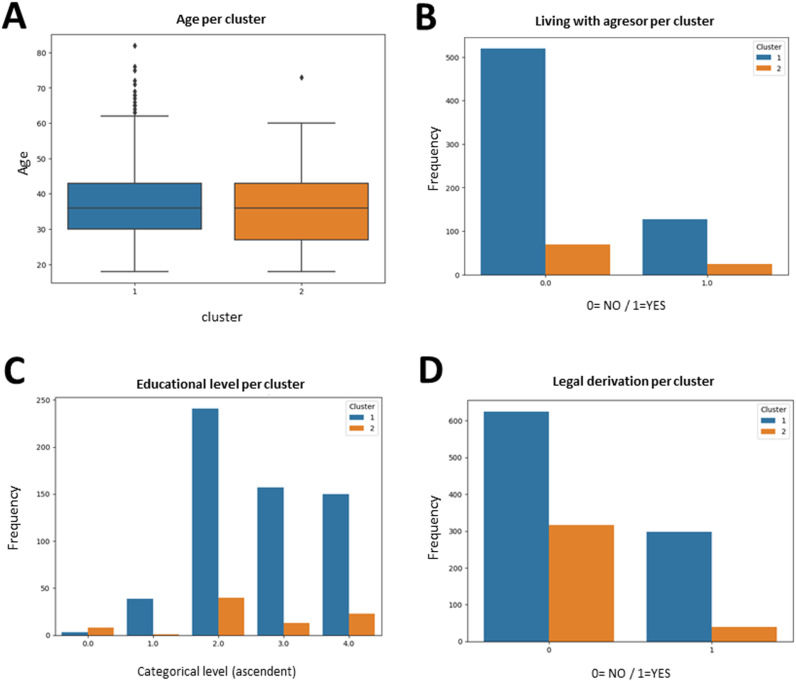
Plots showing patient age distributions, living with aggressor, educational level and legal derivation in each cluster. T test analysis shows no significant differences between means of age: p = 0.756 (A). Chi square association shows no significance (p = 0.21) between living with aggressor state and cluster (B). Educational level frequencies in the two clusters, with 0 indicating no studies, 1: medium, 3: university, and 4: postgraduate studies show significant difference between both clusters, with a p value of 0.037 (C). Distribution of legal derivation (0 = NO, 1 = YES) with respect to the cluster shows significant association between both variables with a p value of 0.0063 (D).

Despite the difference observed in treatment completeness between the two clusters, the age of the users showed no significant differences when analyzing the distribution (Fig. 2A). The age average of the users in cluster 1 was 37.1 years and 36.3 in cluster 2. Other variables such as working outside, medication intake, living with the aggressor and support network were not significant using Chi square analysis to establish associations between variables (Fig. 2B). In cluster 1, 334 women worked outside, while 247 did not (42.4% and 57.4%, respectively). In cluster 2, 40 women did not work outside whereas 20 did (50% and 59%, respectively). In cluster 1, 225 women consumed medication and 281 did not (44.4% and 55.5%). In cluster 2, 41 women consumed no medication and 26 did (61.1% and 38.8%). Regarding living with the aggressor, in cluster 1, 472 women did not live with him (80%) whereas 115 did (19.5%). In cluster 2, there were 66 women who did not live with the aggressor (75.8%) and 21 who did (24.1%) (Fig. 2B).

Support network in cluster 1 was present in 416 cases (73.5%), whereas 150 victims had no support (26.5%). In cluster 2, 53 victims had support network (67,9%), and 25 did not (32%). The level of education in both clusters was mainly intermediate and technical (40.4% and 26.5% in cluster 1 and 47.5% and 15% in cluster 2). There were 8.7% of the users in cluster 2 with no education, while in cluster 1 this population was not observed. The change in educational level is significantly associated with clustering categorization with a p value of 0.03 (Fig. 2C).

Finally, regarding legal referral during or after treatment, cluster 1 contained 569 women who did not request legal orientation and 269 victims who did ask for it (67.9% and 31.2%, respectively). In cluster 2 there were 300 women who did not request legal orientation and 37 victims who did (89% and 11%, respectively). This variable had a significant association with the cluster, with a p-value of 0.006 (Fig. 2D).

#### Treatment completeness prediction according to a XGBoost explainable machine learning classifier.

**Fig 3:**
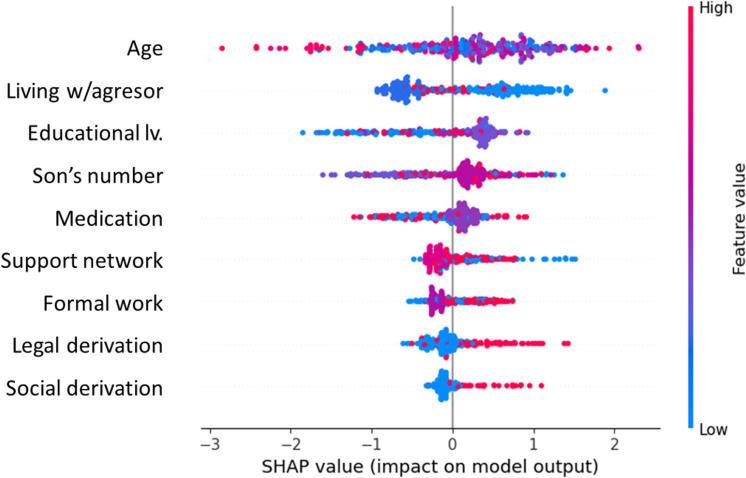
SHAP classification model explicability. This beeswarm graph shows the specific impact on the 0 exit (towards negatives) or 1 (towards positives) of treatment completeness and depicts each parameter value measured in either discrete or continuous variables in color codes. The X-Axis shows each input parameter of the classification model organized according to its absolute impact.

A model for predicting treatment success based on its completeness was developed. The model tested on 25% of the data achieved a maximum accuracy recorded across 50 random states of 0.81. Using the SHAP-based interpretability system, it was determined that the variables with the greatest impact on the classification were age, living with the aggressor, educational level and number of children (Fig 3). On the other hand, the 4 variables with the least impact were having a support network, working outside, legal referral and social referral, but with a categorical behavior of their values with respect to the output of the model (Fig 3).

## Discussion

Despite the significant increase observed in IPV in Chile [25], studies at the national level are scarce. As a consequence, the few existing programs focused on providing the victims with psychological treatment and legal/social orientation lack a local perspective. The latter is crucial, because the social context constitutes a potential risk factor for IPV [16,41]. So, studies focusing on identifying the relevant variables promoting adherence to IPV treatment using quantitative approaches on the victim’s context are essential.

The present research analyzed the variables that contributed to the success of a program designed for IPV victims. An analysis of a database containing 1,279 IPV cases was performed applying classical statistics and artificial intelligence methods. The hierarchical clustering analysis showed that taking a blind data pull two groups appear instantaneously in the analysis cascade. Euclidean distances between each other were high (0.8), which means they are different. Of the two data clusters, one contained 831 users and the other 329 users. The first cluster (cluster 1) corresponded to women who started and completed the treatment with the foundation (one third of it) and the second cluster (cluster 2) corresponded to women who never started the treatment and so did not complete it (none of them).

Categorization of variables presenting a higher impact were analyzed to study the difference between both clusters. The results showed that the most relevant variables for classification were age, living with the aggressor and educational level. Most of the victims asking for help had children (mean, 1.7) and their mean age was 36.6. This suggests that IPV is also present in relationships between young people [14,42], and it may decrease as women grow older [43]. This data is relevant, since it shows that young women are searching for help, as previously described [44,45].

Living with the aggressor is known as a factor increasing IPV exposure, as recently shown by statistical femicide data in Chile and Spain [46,47]. Isolation and the threat context contributed to avoid denunciation [46]. Being ashamed of failure, developing tolerance towards violent behavior and psychological or economic dependence can contribute to this risk [9,48,49].

Education and employment are empowering tools for women, but they do not consistently serve as a protective factor against IPV [50]. the socio-cultural context strongly influences these variables. In patriarchal societies such as the Latin American, women having more education than their partners may have a higher risk of abuse, because gender roles entail that husbands should have more education than their wives [Flake and Forste, 2006]. Consequently, women’s economic empowerment in these contexts leads to an increase in violence, as a response of a threaten to men´s masculinity [26](Saavedra et al., 2022].

However, in the present study, educational level was a significant variable for classification and women with higher educational level were more likely to start and complete the treatment. Moreover, among the crucial factors that made women search for help and finish treatment were working outside and not living with the aggressor. As recently described in [51] Benjumeda-Wynhoven & Yago-Alonso, 2024, women who do not work outside normally consume more medication, as compared to those working outside. So, working outside might provide not just economic independence but also a better health and more self-care possibilities. Economic empowerment and access to resources such as education are crucial factors when reporting violence, which is the first step to break the violence cycle [52][Alvarez et al., 2016]. Programs promoting economic empowerment and education are required to ensure both awareness of the problem along with adherence to IPV treatment programs.

Perceived support is strongly associated with better mental health [53] and lack of support contributes to maintain cohabiting [54]. In the present study, having a support network was not identified as a significant variable for initiating and maintaining treatment. Consistent with this, a study conducted in Nicaragua reported that social support is not always a protective factor for IPV victims [55]; normalization and justification presented by the victim’s environment may be an obstacle to escape IPV, confirming the role of the socio-cultural context in suffering violence [12]. IPV is more common in those countries presenting a patriarchal code that positions women in a submissive condition [37,38][Arce-Rodríguez, 2006, Romero y González, 2017]. Therefore, it is not surprising that Latin America presents one of the highest IPV rates on earth [6,56] [Jaitman and Anauati, 2020, de Souza-Santos et al., 2022]. As described by Alvarez et al., (2016)[52], community and family members sometimes opt to “not get involved” in helping IPV victims, because they are concerned for their own safety or because they are unaware of how to help. These factors can help to understand why social support in Latin America is not always a protective factor for IPV victims. Further analysis on the quality and type of social support is essential in this context.

Analyzing the behavior of the intelligent machine, some of the variables with a significant association lack a unitary impact on the model decision making, whereas others such as age, seem to be very relevant despite presenting no group differences. This can be observed in the SHAP graph (Fig. 3), in which age appears in the first place; observing its values distribution (red, older age and blue, younger) they do not seem to have a categorical distribution with respect to the exit, despite the blue dots present a slight pattern towards exit 1 (treatment completeness). In summary, in order to predict if the victims will complete the treatment, it is not sufficient to observe a single relevant variable; an integrated criterion is required in order to make accurate predictions. A younger patient working outside, not living with the aggressor, having a higher educational level and asking for social or legal orientation has higher probabilities of successfully complete the treatment.

Finally, legal referral was significantly higher in Cluster 1 users. This indicates that the determination to ask for psychological help and legal guidance determined the adherence and success of an IPV intervention program. Despite this, the users did not always finish the psychological treatment, presenting a high dropout rate. Decision to abandon the legal procedure has been shown to correlate with less social support [57].

It is important to emphasize that IPV victims usually come from vulnerable and dysfunctional homes, where violence is normalized [26](Saavedra et al., 2022]. For this reason, they tend to repeat the pattern of violent relationships and develop different psychological disorders including anxiety, depression, and post-traumatic stress disorder (PTSD) [21,44,58][Calvete et al., 2007; Labrador et al., 2010; Guerrero-Vaca et al., 2021]. These mental health alterations make it even more difficult to break the violence cycle. Understanding and treating these patterns and receiving information on their rights and legal terms can open a new door for the victims, who often lack the support of their family and/or friends. All this, together with the normalization of violence against women and gender inequities, explains the lower rate of legal denunciation of IPV victims in Latin America [59][Fernandez-Alonso et al., 2024]. It is worth mentioning that Latin American women usually are less empowered about their rights. In Chile, one in four women report having experienced some type of partner violence (psychological, physical, sexual, or economic), but only 22 percent of female victims of IPV file a formal complaint. The main reasons for not reporting IPV include believing that the violence episodes were not severe, feeling ashamed to report the situation, or believing that reporting is useless ([26]Saavedra et al., 2022]. In most cases, merely informing survivors of IPV about available resources is crucial for help seeking and for intervention [52][Alvarez et al., 2016].

Dropout refers to the interruption of the therapeutic process before or during its formal development and is a common problem in therapeutic care [60]. Worldwide, the dropout rate in psychological therapy is between 25 and 50%. Previous studies report that 26–50% of clients abandon psychiatric and psychological care after the first session [61]. The reasons for dropout can be diverse and range from the therapist, their capacity and personality the institution or the user´s specific economic, work or family situation [62]. In relation to dropping out of treatment in IPV victims, to date the literature investigating the reasons is scarce, despite abandoning is a common circumstance. Previous studies defend a low correlation between the reasons for dropout and the therapist/institution. The attributed causes are mainly related to an interaction between factors associated with the users´ health aspects, fear of the aggressor, working outside, caring responsibilities, distance and lack of interest [60].

Previous studies defend that some of the reasons for abandoning treatment in IPV victims have their roots in cultural aspects that maintain a system of gender inequality (overload of domestic tasks, economic dependence, subordination and consequent lack of self-esteem and self-worth). In this sense, women dropout the treatment in order to continue with their daily activities, considered as the most important in their lives according to cultural mandates prevailing in Latin American societies [14]. In this countries, women normalize discomfort and inequality as part of their “condition”, accepting the context in which they live as hostile and violent [4,12]. This normalization would increase treatment dropout, since the victim believes that the treatment is no longer necessary and would only resume it when the situation becomes unsustainable [63]. According to Leonor Walker’s model [64], IPV is a cyclical process divided into 3 stages: the accumulation of tension, in which there is a gradual increase in tension, with hostile acts of the man towards the woman like shouting or getting angry. This stage is followed by aggression, which along with psychological violence usually includes physical and/or sexual violence. The aggressor blames the victim for what happened, thus justifying aggression. The phase that follows aggression is the honeymoon or reconciliation stage, in which the aggressor promises that he will change. Intermittentness of these stages leads to emotional dependence and to both physical and psychological devastation. On the other hand, the transtheoretical model of change by Prochaska and DiClemente defends the existence of different phases in the process of becoming aware and abandoning a violent relationship [65]. These phases are precontemplation, contemplation, preparation, action, maintenance and relapse. According to this model, the change does not occur in a linear way, but is rather circular, crossing different phases. During the process, the victim may become stagnant and even retreat. Therefore, it is crucial to know in which of these stages is the IPV victim, since her level of awareness and firmness to leave the relationship will differ depending on the process stage. Also, it is essential to understand that cultural norms strongly influence treatment adherence in terms of the expectations of how a “good” woman should behave, emphasizing the position of a self-sacrificing woman who is devoted to her family. Gender based norms reinforce male authority and superiority over females in most Latin American countries [36][Flake and Forste, 2006]. In this context, machismo is often used to describe Latino masculinity, and refers to the cultural expectation that males must be masculine, strong, and sexually aggressive [39][James, 2010; Malonda et al., 2022]. To mitigate these barriers in IPV intervention programs, education plays a key role; it is crucial to promote equal access to education and develop prevention strategies at schools, promoting initiatives that address gender inequalities from a young age. Children should learn to build gender relations based on mutual respect and non-violence [26](Saavedra et al., 2022]. As for IPV victims, previous studies have shown that group sessions increase self-awareness, feelings of empowerment, peer support, and self-efficacy, which are all critical aspects for behavior modification [52][Alvarez et al., 2016]. These strategies may help to mitigate cultural norms, improving intervention programs´ adherence.

The value of this study relies on the relevance of being able to predict the success of a treatment focused on IPV victims in the Chilean context. The results demonstrate that desertion is associated with a group of users who share certain characteristics such as age, living with the aggressor or having a lower educational level. Also, this research shows that AI tools such as clusterization and predictor modeling are extremely useful instruments for both designing and adapting IPV treatments and public policies focused on women health.

## Conclusions

Using an intelligent machine classification analysis, this study demonstrates that, in order to predict if IPV victims will complete the treatment, it is not sufficient to observe a single relevant variable. However, an integrated criterion is required in order to make accurate predictions. A younger patient working outside, not living with the aggressor, having a higher educational level and asking for social or legal orientation has higher probabilities of successfully complete the treatment.

In line with this, legal derivation appeared as a significant variable that could be used as treatment success predictor. It can be concluded that given the cyclic process of IPV, when the victim searches for legal orientation she is aware of the problem and is willing to abandon the relationship. In doing so, she is focused on organizing legal aspects of the separation process such as children custody, alimony and eventually, complaint for violence.

This research demonstrates that predicting the success of the treatment based on completeness using a machine learning model is possible due to the presence of two patient groups in the data, as demonstrated by the two clusters and by the descriptive statistics.

Designing appropriate programs for women suffering gender violence is essential to help them reorient their lifes and improve well-being. In this sense, knowing the psychological functioning of violence victims and the issues that contribute to maintain IPV is critical. In order to avoid treatment abandonment or not adherence, understanding how these women internalize and normalize violence patterns and the context they are immersed is fundamental for re-victimization prevention.

To conclude, using AI tools such as clusterization and predictive modeling are very useful for data organization and variable analysis and should be further applied in research focused on making predictions relating IPV to different socio cultural aspects, such as female unemployment or mental health.

### Study limitations

This study has a few limitations. First, further research should delve into the type of violence experienced by IPV survivors to correlate this information with variables such as working, medication intake and living with the aggressor. The specific type of social support received and of medication intake is also relevant and should be deeper analyzed.

Also, the results of this study may be affected by the social desirability of the participants, especially in those variables asking for medication intake and living with the aggressor. Qualitative data may contribute to solve this, providing more information on the victim´s context.

On the other hand, the model used for the classification uses a gradient boosting structure, and although its accuracy performance was 0.81, it is possible to increase this performance in the future with more complex models based on Deep Learning and/or increasing the volume of input data.

Finally, it is important to note that the analyzed sample is not representative of all women experiencing IPV in Chile, but only of those who have searched for help and are already prepared to break the cycle of violence, despite a big amount of the sample abandoning the process.

Despite these limitations, this study significantly contributes to the literature expanding knowledge on the factors that may contribute to seek for assistance and further adhere and complete a psychological program aimed at IPV victims.

## Supporting information

S1 SirusTextFile. Refers to SIRUS_Plos3.html. Refers to programming.
(HTML)

S2 DataBase Sirus Plos2.xlsx. Refers to excel data base.
(XLSX)

S3 DataBase SirusPlos.xlsx. Referes to excel data base.
(XLSX)
