# Correction: Analysis of IPV success treatment from an AI approach

**DOI:** 10.1371/journal.pone.0332535

**Published:** 2025-09-15

**Authors:** Isabel María Benjumeda Wynhoven, Claudio Córdova Lepe

There is an error in affiliation 2 for author Claudio Córdova Lepe. The correct affiliation 2 is: Centro Interdisciplinario de Investigación Biomédica e Ingeniería para la Salud MEDING, Universidad de Valparaiso, Valparaíso, Chile.
