# Supplementary material for: Analysis of IPV success treatment from an AI approach
Source: PLoS One. 2025 Jun 3;20(6):e0323945. doi: 10.1371/journal.pone.0323945 (PMC12132942; doi:10.1371/journal.pone.0323945)
Supplement: S1 — Refers to SIRUS_Plos3.html. Refers to programming. (HTML) [file pone.0323945.s001.html]

SIRUS


 


In [1]:

```
import psutil

# Obtener información sobre el uso de memoria
mem = psutil.virtual_memory()

# Imprimir el uso de memoria en Jupyter Notebook
print(f"Uso de memoria: {mem.used / 1024**3:.2f} GB")
```

```
Uso de memoria: 10.38 GB
```

In [2]:

```
import tensorflow as tf
import pynvml

# Verificar si TensorFlow está utilizando la GPU
if tf.test.is_gpu_available():
    print("TensorFlow está utilizando la GPU")
    
    # Inicializar la biblioteca pynvml
    pynvml.nvmlInit()
    
    # Obtener el número de dispositivos GPU
    num_gpus = pynvml.nvmlDeviceGetCount()
    
    # Iterar sobre los dispositivos GPU
    for i in range(num_gpus):
        # Obtener el identificador del dispositivo
        handle = pynvml.nvmlDeviceGetHandleByIndex(i)
        # Obtener el nombre completo de la GPU
        gpu_name = pynvml.nvmlDeviceGetName(handle)
        print("Nombre de la GPU:", gpu_name)
        
    # Finalizar la biblioteca pynvml
    pynvml.nvmlShutdown()
else:
    print("TensorFlow no está utilizando la GPU")
```

```
WARNING:tensorflow:From C:\Users\claud\AppData\Local\Temp\ipykernel_16936\733174528.py:5: is_gpu_available (from tensorflow.python.framework.test_util) is deprecated and will be removed in a future version.
Instructions for updating:
Use `tf.config.list_physical_devices('GPU')` instead.
TensorFlow está utilizando la GPU
Nombre de la GPU: NVIDIA GeForce GTX 1650 with Max-Q Design
```

In [3]:

```
# Obtiene la versión de CUDA a través de la configuración de TensorFlow
cuda_version = tf.sysconfig.get_build_info()['cuda_version']

print('Versión de CUDA:', cuda_version)
```

```
Versión de CUDA: 64_112
```

In [4]:

```
import pandas as pd
import matplotlib.pyplot as plt
from itertools import combinations
from sklearn.linear_model import LinearRegression
from sklearn.ensemble import RandomForestRegressor
from sklearn.metrics import r2_score, mean_squared_error
from sklearn.metrics import r2_score
import itertools
import sys
import numpy as np
from sklearn.metrics import r2_score, mean_squared_error
import matplotlib.pyplot as plt
import tensorflow as tf
import pynvml
import pickle
from sklearn import tree
import graphviz
from sklearn.cluster import KMeans
from lifelines import KaplanMeierFitter
import scipy.stats as stats
from statsmodels.stats.multicomp import pairwise_tukeyhsd
import pingouin as pg
from lifelines.statistics import logrank_test
from lifelines.statistics import pairwise_logrank_test
```

In [5]:

```
# Ajustar la configuración de visualización de pandas
pd.set_option('display.max_rows', 50)
pd.set_option('display.max_columns', 50)
```

In [6]:

```
# Cargar el dataframe desde un archivo Excel
df = pd.read_excel('SIRUS2.xlsx', sheet_name= 'Sheet1')  # Reemplaza 'ruta_del_archivo.xlsx' con la ruta correcta de tu archivo
# Convierte el DataFrame de Pandas a un DataFrame de Dask

df
```

```
C:\Users\claud\anaconda3\envs\tf\lib\site-packages\openpyxl\worksheet\_reader.py:329: UserWarning: Unknown extension is not supported and will be removed
  warn(msg)
C:\Users\claud\anaconda3\envs\tf\lib\site-packages\openpyxl\worksheet\_reader.py:329: UserWarning: Conditional Formatting extension is not supported and will be removed
  warn(msg)
```

Out[6]:

|  | Fecha Solicitud | Edad | Hijos\_IA | Trabaja fuera | Medicamentos\_IA | Vive con agresor | Nivel de estudios | Tiene red apoyo | Derivacion social | Derivacion legal | Estado ficha | OCC\_IA | OCT\_IA |
| --- | --- | --- | --- | --- | --- | --- | --- | --- | --- | --- | --- | --- | --- |
| 0 | 2021-09-09 21:21:00 | 44 | NaN | NaN | NaN | NaN | NaN | NaN | 0 | 0 | 2.0 | 0.0 | 2.0 |
| 1 | 2021-09-09 14:39:00 | 46 | NaN | NaN | NaN | NaN | NaN | NaN | 0 | 0 | 2.0 | 1.0 | 2.0 |
| 2 | 2021-09-08 23:03:00 | 41 | 4.0 | 1.0 | 0.0 | 0.0 | 4.0 | 1.0 | 0 | 1 | 2.0 | NaN | NaN |
| 3 | 2021-09-08 20:03:00 | 43 | 2.0 | 1.0 | 1.0 | 0.0 | 3.0 | NaN | 0 | 1 | 2.0 | 1.0 | 1.0 |
| 4 | 2021-09-09 23:47:00 | 53 | NaN | NaN | NaN | NaN | NaN | NaN | 0 | 0 | 2.0 | 1.0 | 1.0 |
| ... | ... | ... | ... | ... | ... | ... | ... | ... | ... | ... | ... | ... | ... |
| 1106 | 2023-07-12 11:40:00 | 35 | NaN | NaN | NaN | NaN | NaN | NaN | 0 | 0 | NaN | NaN | NaN |
| 1107 | 2023-07-18 17:19:00 | 38 | NaN | NaN | NaN | NaN | NaN | NaN | 0 | 0 | NaN | NaN | NaN |
| 1108 | 2023-07-12 13:34:00 | 36 | NaN | NaN | NaN | NaN | NaN | NaN | 0 | 0 | NaN | NaN | NaN |
| 1109 | 2023-07-15 21:07:00 | 41 | NaN | NaN | NaN | NaN | NaN | NaN | 0 | 0 | NaN | NaN | NaN |
| 1110 | 2023-07-20 00:49:00 | 34 | 1.0 | 0.0 | 0.0 | 0.0 | 2.0 | 1.0 | 0 | 1 | 1.0 | NaN | NaN |

1111 rows × 13 columns

In [36]:

```
# Ajustar la configuración de visualización de pandas
#pd.set_option('display.max_rows', None)
#pd.set_option('display.max_columns', None)

# Obtener información del tipo de dato de todas las columnas
info_completa = df.dtypes

# Descartar la columna 'Fecha Solicitud'
df2 = df.drop(['Fecha Solicitud'], axis=1)


print(info_completa)
```

```
Fecha Solicitud      datetime64[ns]
Edad                          int64
Hijos_IA                    float64
Trabaja fuera               float64
Medicamentos_IA             float64
Vive con agresor            float64
Nivel de estudios           float64
Tiene red apoyo             float64
Derivacion social             int64
Derivacion legal              int64
Estado ficha                float64
OCC_IA                      float64
OCT_IA                      float64
dtype: object
```

In [37]:

```
from scipy.cluster.hierarchy import dendrogram, linkage
from scipy.spatial.distance import jaccard

# Define una función de distancia personalizada
def distancia_jaccard(u, v):
    return jaccard(u.astype(bool), v.astype(bool))

# Calcular el clustering jerárquico usando la función de distancia personalizada y el método de enlace completo
Z = linkage(df2.to_numpy(), metric=distancia_jaccard, method='complete')

# Crear el dendrograma
import matplotlib.pyplot as plt
plt.figure(figsize=(25, 10))
plt.title('Dendrograma jerárquico para el Clustering')
plt.xlabel('Índice de la Muestra')
plt.ylabel('Distancia')
dendrogram(Z, leaf_rotation=90., leaf_font_size=0.01)
plt.show()
```

In [ ]:

```
# Aplicar codificación One-Hot a la columna 'Comuna'
#df2 = pd.get_dummies(df2, columns=['Comuna'])

# Ahora puedes continuar con el clustering jerárquico
from scipy.cluster.hierarchy import dendrogram, linkage
Z = linkage(df2, 'ward')

# Crear el dendrograma
import matplotlib.pyplot as plt
plt.figure(figsize=(25, 10))
plt.title('Dendrograma jerárquico para el Clustering')
plt.xlabel('Índice de la Muestra')
plt.ylabel('Distancia')
dendrogram(Z, leaf_rotation=90., leaf_font_size=0.01)
plt.show()
```

In [65]:

```
from scipy.cluster.hierarchy import dendrogram, linkage, fcluster
import matplotlib.pyplot as plt

# Cortar el dendrograma en la distancia especificada y asignar las etiquetas a los datos
df2['Cluster'] = fcluster(Z, t=200, criterion='distance')
```

In [38]:

```
from scipy.cluster.hierarchy import dendrogram, linkage, fcluster
import matplotlib.pyplot as plt

# Calcular el clustering jerárquico usando la función de distancia personalizada y el método de enlace completo
Z = linkage(df2.to_numpy(), metric=distancia_jaccard, method='complete')

# Crear el dendrograma
plt.figure(figsize=(25, 10))
plt.title('Dendrograma jerárquico para el Clustering')
plt.xlabel('Índice de la Muestra')
plt.ylabel('Distancia')
dendrogram(Z, leaf_rotation=90., leaf_font_size=0.01)

# Cortar el dendrograma en la distancia especificada y asignar las etiquetas a los datos
distancia_corte = 0.8
clusters = fcluster(Z, t=distancia_corte, criterion='distance')
df2['Cluster'] = clusters

# Mostrar el dendrograma con el corte
plt.axhline(y=distancia_corte, color='r', linestyle='--')
plt.show()
```

In [39]:

```
# Contabilizar los valores en la columna 'Cluster'
conteo = df2['Cluster'].value_counts()

# Imprimir el conteo
print(conteo)
```

```
Cluster
1    792
3    314
2      5
Name: count, dtype: int64
```

In [40]:

```
import pandas as pd
import matplotlib.pyplot as plt
import seaborn as sns
from scipy.stats import f_oneway

# Box plot de Edad agrupado por Cluster usando seaborn
plt.figure(figsize=(8, 6))
sns.boxplot(x='Cluster', y='Edad', data=df2)
plt.title('Box Plot de Edad por Cluster')
plt.xlabel('Cluster')
plt.ylabel('Edad')
plt.show()

# Realizar la prueba ANOVA
cluster_groups = [group['Edad'] for name, group in df2.groupby('Cluster')]
f_statistic, p_value = f_oneway(*cluster_groups)

print("\nResultado de la prueba ANOVA:")
print(f"F-statistic: {f_statistic}")
print(f"P-value: {p_value}")

# Interpretación del resultado
alpha = 0.05  # Nivel de significancia


if p_value < alpha:
    print("\nConclusion: Hay diferencias significativas en las edades entre los grupos de Clusters.")
else:
    print("\nConclusion: No hay diferencias significativas en las edades entre los grupos de Clusters.")
```

```
Resultado de la prueba ANOVA:
F-statistic: 1.0104649693668504
P-value: 0.3643848926480682

Conclusion: No hay diferencias significativas en las edades entre los grupos de Clusters.
```

In [41]:

```
from scipy.stats import chi2_contingency

# Crear una tabla de contingencia para la columna "Hijos" y el "Cluster"
contingency_table_hijos = pd.crosstab(df2['Hijos_IA'], df2['Cluster'])

# Realizar la prueba de chi-cuadrado para la columna "Hijos"
chi2_stat_hijos, p_value_hijos, dof_hijos, expected_hijos = chi2_contingency(contingency_table_hijos)

print("\nResultado de la prueba de chi-cuadrado para Hijos:")
print(f"Chi-square statistic: {chi2_stat_hijos}")
print(f"P-value: {p_value_hijos}")

# Gráfico de barras para la columna "Hijos" por Cluster
plt.figure(figsize=(8, 6))
sns.countplot(x='Hijos_IA', hue='Cluster', data=df2)
plt.title('Frecuencia de Hijos por Cluster')
plt.xlabel('Hijos')
plt.ylabel('Frecuencia')
plt.show()

# Interpretación del resultado para Hijos
if p_value_hijos < alpha:
    print("\nConclusion: Existe una asociación significativa entre la variable 'Hijos' y el 'Cluster'.")
else:
    print("\nConclusion: No existe una asociación significativa entre la variable 'Hijos' y el 'Cluster'.")
```

```
Resultado de la prueba de chi-cuadrado para Hijos:
Chi-square statistic: 60.157738245633006
P-value: 1.9293897203037516e-06
```

```
Conclusion: Existe una asociación significativa entre la variable 'Hijos' y el 'Cluster'.
```

In [42]:

```
from scipy.stats import chi2_contingency

# Crear una tabla de contingencia para la columna "Trabaja fuera" y el "Cluster"
contingency_table_trabaja_fuera = pd.crosstab(df2['Trabaja fuera'], df2['Cluster'])

# Realizar la prueba de chi-cuadrado para la columna "Trabaja fuera"
chi2_stat_trabaja_fuera, p_value_trabaja_fuera, dof_trabaja_fuera, expected_trabaja_fuera = chi2_contingency(contingency_table_trabaja_fuera)

print("\nResultado de la prueba de chi-cuadrado para Trabaja Fuera:")
print(f"Chi-square statistic: {chi2_stat_trabaja_fuera}")
print(f"P-value: {p_value_trabaja_fuera}")


# Gráfico de barras para la columna "Trabaja fuera" por Cluster
plt.figure(figsize=(8, 6))
sns.countplot(x='Trabaja fuera', hue='Cluster', data=df2)
plt.title('Distribución de Trabaja Fuera por Cluster')
plt.xlabel('Trabaja Fuera')
plt.ylabel('Frecuencia')
plt.show()

# Interpretación del resultado para Trabaja Fuera
alpha = 0.05

if p_value_trabaja_fuera < alpha:
    print("\nConclusion: Existe una asociación significativa entre la variable 'Trabaja Fuera' y el 'Cluster'.")
else:
    print("\nConclusion: No existe una asociación significativa entre la variable 'Trabaja Fuera' y el 'Cluster'.")
```

```
Resultado de la prueba de chi-cuadrado para Trabaja Fuera:
Chi-square statistic: 19.676595116696777
P-value: 5.3368089882924056e-05
```

```
Conclusion: Existe una asociación significativa entre la variable 'Trabaja Fuera' y el 'Cluster'.
```

In [43]:

```
# Crear una tabla de contingencia para la columna "Nivel de estudios" y el "Cluster"
contingency_table_nivel_estudios = pd.crosstab(df2['Nivel de estudios'], df2['Cluster'])

# Realizar la prueba de chi-cuadrado para la columna "Nivel de estudios"
chi2_stat_nivel_estudios, p_value_nivel_estudios, dof_nivel_estudios, expected_nivel_estudios = chi2_contingency(contingency_table_nivel_estudios)

print("\nResultado de la prueba de chi-cuadrado para Nivel de Estudios:")
print(f"Chi-square statistic: {chi2_stat_nivel_estudios}")
print(f"P-value: {p_value_nivel_estudios}")

# Gráfico de barras para la columna "Nivel de estudios" por Cluster
plt.figure(figsize=(8, 6))
sns.countplot(x='Nivel de estudios', hue='Cluster', data=df2)
plt.title('Distribución de Nivel de Estudios por Cluster')
plt.xlabel('Nivel de Estudios')
plt.ylabel('Frecuencia')
plt.show()

# Interpretación del resultado para Nivel de Estudios
if p_value_nivel_estudios < alpha:
    print("\nConclusion: Existe una asociación significativa entre la variable 'Nivel de Estudios' y el 'Cluster'.")
else:
    print("\nConclusion: No existe una asociación significativa entre la variable 'Nivel de Estudios' y el 'Cluster'.")
```

```
Resultado de la prueba de chi-cuadrado para Nivel de Estudios:
Chi-square statistic: 323.0502082603975
P-value: 5.072221611555396e-65
```

```
Conclusion: Existe una asociación significativa entre la variable 'Nivel de Estudios' y el 'Cluster'.
```

In [44]:

```
# Crear una tabla de contingencia para la columna "Tiene red apoyo" y el "Cluster"
contingency_table_red_apoyo = pd.crosstab(df2['Tiene red apoyo'], df2['Cluster'])

# Realizar la prueba de chi-cuadrado para la columna "Tiene red apoyo"
chi2_stat_red_apoyo, p_value_red_apoyo, dof_red_apoyo, expected_red_apoyo = chi2_contingency(contingency_table_red_apoyo)

print("\nResultado de la prueba de chi-cuadrado para Tiene Red de Apoyo:")
print(f"Chi-square statistic: {chi2_stat_red_apoyo}")
print(f"P-value: {p_value_red_apoyo}")

# Gráfico de barras para la columna "Tiene red apoyo" por Cluster
plt.figure(figsize=(8, 6))
sns.countplot(x='Tiene red apoyo', hue='Cluster', data=df2)
plt.title('Distribución de Tiene Red de Apoyo por Cluster')
plt.xlabel('Tiene Red de Apoyo')
plt.ylabel('Frecuencia')
plt.show()

# Interpretación del resultado para Tiene Red de Apoyo
if p_value_red_apoyo < alpha:
    print("\nConclusion: Existe una asociación significativa entre la variable 'Tiene Red de Apoyo' y el 'Cluster'.")
else:
    print("\nConclusion: No existe una asociación significativa entre la variable 'Tiene Red de Apoyo' y el 'Cluster'.")
```

```
Resultado de la prueba de chi-cuadrado para Tiene Red de Apoyo:
Chi-square statistic: 16.413750465652257
P-value: 0.000272771739157208
```

```
Conclusion: Existe una asociación significativa entre la variable 'Tiene Red de Apoyo' y el 'Cluster'.
```

In [45]:

```
# Crear una tabla de contingencia para la columna "Medicamentos" y el "Cluster"
contingency_table_medicamentos = pd.crosstab(df2['Medicamentos_IA'], df2['Cluster'])

# Realizar la prueba de chi-cuadrado para la columna "Medicamentos"
chi2_stat_medicamentos, p_value_medicamentos, dof_medicamentos, expected_medicamentos = chi2_contingency(contingency_table_medicamentos)

print("\nResultado de la prueba de chi-cuadrado para Uso de Medicamentos:")
print(f"Chi-square statistic: {chi2_stat_medicamentos}")
print(f"P-value: {p_value_medicamentos}")

# Gráfico de barras para la columna "Medicamentos" por Cluster
plt.figure(figsize=(8, 6))
sns.countplot(x='Medicamentos_IA', hue='Cluster', data=df2)
plt.title('Distribución de Uso de Medicamentos por Cluster')
plt.xlabel('Uso de Medicamentos')
plt.ylabel('Frecuencia')
plt.show()


# Interpretación del resultado para Uso de Medicamentos
if p_value_medicamentos < alpha:
    print("\nConclusion: Existe una asociación significativa entre la variable 'Uso de Medicamentos' y el 'Cluster'.")
else:
    print("\nConclusion: No existe una asociación significativa entre la variable 'Uso de Medicamentos' y el 'Cluster'.")
```

```
Resultado de la prueba de chi-cuadrado para Uso de Medicamentos:
Chi-square statistic: 18.025669587893198
P-value: 0.00012183598609980331
```

```
Conclusion: Existe una asociación significativa entre la variable 'Uso de Medicamentos' y el 'Cluster'.
```

In [46]:

```
# Crear una tabla de contingencia para la columna "Vive con Agresor" y el "Cluster"
contingency_table_vive_agresor = pd.crosstab(df2['Vive con agresor'], df2['Cluster'])

# Realizar la prueba de chi-cuadrado para la columna "Vive con Agresor"
chi2_stat_vive_agresor, p_value_vive_agresor, dof_vive_agresor, expected_vive_agresor = chi2_contingency(contingency_table_vive_agresor)

print("\nResultado de la prueba de chi-cuadrado para Vive con Agresor:")
print(f"Chi-square statistic: {chi2_stat_vive_agresor}")
print(f"P-value: {p_value_vive_agresor}")

# Gráfico de barras para la columna "vive con agresor" por Cluster
plt.figure(figsize=(8, 6))
sns.countplot(x='Vive con agresor', hue='Cluster', data=df2)
plt.title('Distribución de Vivienda con Agresor por Cluster')
plt.xlabel('Vive con Agresor')
plt.ylabel('Frecuencia')
plt.show()


# Interpretación del resultado para Vive con Agresor
if p_value_vive_agresor < alpha:
    print("\nConclusion: Existe una asociación significativa entre la variable 'Vive con Agresor' y el 'Cluster'.")
else:
    print("\nConclusion: No existe una asociación significativa entre la variable 'Vive con Agresor' y el 'Cluster'.")
```

```
Resultado de la prueba de chi-cuadrado para Vive con Agresor:
Chi-square statistic: 1.9335210975819872
P-value: 0.3803130503161215
```

```
Conclusion: No existe una asociación significativa entre la variable 'Vive con Agresor' y el 'Cluster'.
```

In [47]:

```
# Crear una tabla de contingencia para la columna "Vive con Agresor" y el "Cluster"
contingency_table_vive_agresor = pd.crosstab(df2['Derivacion social'], df2['Cluster'])

# Realizar la prueba de chi-cuadrado para la columna "Vive con Agresor"
chi2_stat_vive_agresor, p_value_vive_agresor, dof_vive_agresor, expected_vive_agresor = chi2_contingency(contingency_table_vive_agresor)

print("\nResultado de la prueba de chi-cuadrado para Vive con Agresor:")
print(f"Chi-square statistic: {chi2_stat_vive_agresor}")
print(f"P-value: {p_value_vive_agresor}")

# Gráfico de barras para la columna "vive con agresor" por Cluster
plt.figure(figsize=(8, 6))
sns.countplot(x='Derivacion social', hue='Cluster', data=df2)
plt.title('Distribución de Derivacion social por Cluster')
plt.xlabel('Derivacion social')
plt.ylabel('Frecuencia')
plt.show()


# Interpretación del resultado para Vive con Agresor
if p_value_vive_agresor < alpha:
    print("\nConclusion: Existe una asociación significativa entre la variable 'Derivacion social' y el 'Cluster'.")
else:
    print("\nConclusion: No existe una asociación significativa entre la variable 'Derivacion social' y el 'Cluster'.")
```

```
Resultado de la prueba de chi-cuadrado para Vive con Agresor:
Chi-square statistic: 1.7803703178183496
P-value: 0.41057972322010916
```

```
Conclusion: No existe una asociación significativa entre la variable 'Derivacion social' y el 'Cluster'.
```

In [48]:

```
# Crear una tabla de contingencia para la columna "Vive con Agresor" y el "Cluster"
contingency_table_vive_agresor = pd.crosstab(df2['Derivacion legal'], df2['Cluster'])

# Realizar la prueba de chi-cuadrado para la columna "Vive con Agresor"
chi2_stat_vive_agresor, p_value_vive_agresor, dof_vive_agresor, expected_vive_agresor = chi2_contingency(contingency_table_vive_agresor)

print("\nResultado de la prueba de chi-cuadrado para Vive con Agresor:")
print(f"Chi-square statistic: {chi2_stat_vive_agresor}")
print(f"P-value: {p_value_vive_agresor}")

# Gráfico de barras para la columna "vive con agresor" por Cluster
plt.figure(figsize=(8, 6))
sns.countplot(x='Derivacion legal', hue='Cluster', data=df2)
plt.title('Distribución de Derivacion legal por Cluster')
plt.xlabel('Derivacion legal')
plt.ylabel('Frecuencia')
plt.show()


# Interpretación del resultado para Vive con Agresor
if p_value_vive_agresor < alpha:
    print("\nConclusion: Existe una asociación significativa entre la variable 'Derivacion legal' y el 'Cluster'.")
else:
    print("\nConclusion: No existe una asociación significativa entre la variable 'Derivacion legal' y el 'Cluster'.")
```

```
Resultado de la prueba de chi-cuadrado para Vive con Agresor:
Chi-square statistic: 25.164650651622928
P-value: 3.432144320443339e-06
```

```
Conclusion: Existe una asociación significativa entre la variable 'Derivacion legal' y el 'Cluster'.
```

In [49]:

```
# Obtener información del tipo de dato de todas las columnas
info_completa = df2.dtypes

print(info_completa)
```

```
Edad                   int64
Hijos_IA             float64
Trabaja fuera        float64
Medicamentos_IA      float64
Vive con agresor     float64
Nivel de estudios    float64
Tiene red apoyo      float64
Derivacion social      int64
Derivacion legal       int64
Estado ficha         float64
OCC_IA               float64
OCT_IA               float64
Cluster                int32
dtype: object
```

In [50]:

```
df3 = df2.drop(['Cluster'], axis=1)
info_completa = df3.dtypes

print(info_completa)
```

```
Edad                   int64
Hijos_IA             float64
Trabaja fuera        float64
Medicamentos_IA      float64
Vive con agresor     float64
Nivel de estudios    float64
Tiene red apoyo      float64
Derivacion social      int64
Derivacion legal       int64
Estado ficha         float64
OCC_IA               float64
OCT_IA               float64
dtype: object
```

In [85]:

```
import matplotlib.pyplot as plt
from sklearn.tree import plot_tree
from sklearn.metrics import precision_score, recall_score

# Manejar NaN (por ejemplo, rellenar con la media)
df3 = df3.fillna(df3.mean())

# Definir X (features) y y (etiquetas)
X = df3.drop('Derivacion legal', axis=1)
y = df3['Derivacion legal']

# Dividir los datos en conjuntos de entrenamiento y prueba
X_train, X_test, y_train, y_test = train_test_split(X, y, test_size=0.25, random_state=3)

# Inicializar y entrenar el clasificador (por ejemplo, usando un RandomForestClassifier)
clf = RandomForestClassifier()
clf.fit(X_train, y_train)

# Predecir en el conjunto de prueba
y_pred = clf.predict(X_test)

# Calcular la precisión del clasificador
accuracy = accuracy_score(y_test, y_pred)
print(f'Precisión del clasificador: {accuracy}')
```

```
Precisión del clasificador: 0.7697841726618705
```

In [86]:

```
import shap

# Inicializar el explorador SHAP
explainer = shap.Explainer(clf, X_train)

# Calcular las explicaciones para un conjunto de datos de prueba
shap_values = explainer.shap_values(X_test)

# Generar un resumen de las contribuciones de características para todas las clases
shap.summary_plot(shap_values, X_test, feature_names=X.columns)
```

In [82]:

```
from xgboost import XGBClassifier
from sklearn.metrics import accuracy_score

# Manejar NaN (por ejemplo, rellenar con la media)
df3 = df3.fillna(df3.mean())

# Definir X (features) y y (etiquetas)
X = df3.drop('Derivacion legal', axis=1)
y = df3['Derivacion legal']

# Dividir los datos en conjuntos de entrenamiento y prueba
X_train, X_test, y_train, y_test = train_test_split(X, y, test_size=0.25, random_state=13)

# Inicializar y entrenar el clasificador (usando XGBoost)
clf2 = XGBClassifier()
clf2.fit(X_train, y_train)

# Predecir en el conjunto de prueba
y_pred = clf2.predict(X_test)

# Calcular la precisión del clasificador
accuracy = accuracy_score(y_test, y_pred)
print(f'Precisión del clasificador: {accuracy}')
```

```
Precisión del clasificador: 0.7517985611510791
```

In [83]:

```
import shap

# Inicializar el explorador SHAP
explainer = shap.Explainer(clf2, X_train)

# Calcular las explicaciones para un conjunto de datos de prueba
shap_values = explainer.shap_values(X_test)

# Generar un resumen de las contribuciones de características para todas las clases
shap.summary_plot(shap_values, X_test, feature_names=X.columns)
```

```
No data for colormapping provided via 'c'. Parameters 'vmin', 'vmax' will be ignored
```

In [ ]:

```

```
